# Improving thermo-tolerance of *Saccharomyces cerevisiae* by precise regulation of the expression of small HSP†

**DOI:** 10.1039/d3ra05216h

**Published:** 2023-12-12

**Authors:** Mei-ling Zhang, Hui Zhang, Ya-xin He, Zhao-hui Wu, Ke Xu

**Affiliations:** a Department of Life Science, Tangshan Normal University Tangshan 063000 PR China; b Department of Chemical Engineering, Key Lab for Industrial Biocatalysis, Ministry of Education, Tsinghua University Beijing 100084 PR China xuke528@tsinghua.edu.cn

## Abstract

The level of heat resistance in microbial cells is an important factor in determining the energy consumption and product synthesis efficiency of fermentation processes. Current research generally believes that heat shock proteins (HSPs) are the most closely related functional molecules to heat resistance inside cells. They can stabilize cell structures and allow cells to perform their normal physiological functions. Based on our previous transcriptome data, this study applies synthetic biology methods to validate the functionality of heat-resistant elements. The researchers introduced gene circuits expressing small HSPs (*sHSP-HB8*, *HSP12*, *HSP26*, *HSP30*, *HSP42*, and *ibpa-MB4*) with different promoter strengths (*TDH3*p, *YNL247w*p) into *Saccharomyces cerevisiae* strains for functional verification. All engineered strains, with the exception of No. 3 and No. 8, demonstrated a significantly higher growth rate and cell viability at 42 °C. Among them, No. 7 (*YNL247w*p-*HSP12-SLM5*t) and No. 11 (*YNL247w*p-*sHSP-HB8-SLM5*t), the two best performing engineered strains, exhibited a 19.8% and 17.2% increase in cell density, respectively, compared to the control strain. Additionally, the analysis of pyruvate kinase (PK) and malate dehydrogenase (MDH) enzyme activities indicated that the engineered strains enhanced protein quality at higher temperatures. The research methods and ideas presented in this paper have significant scientific reference value for exploring and applying other stress-resistant gene circuits.

## Abstract 2

The level of heat resistance in microbial cells is an important factor in determining the energy consumption and product synthesis efficiency of fermentation processes.

## Graphical abstract

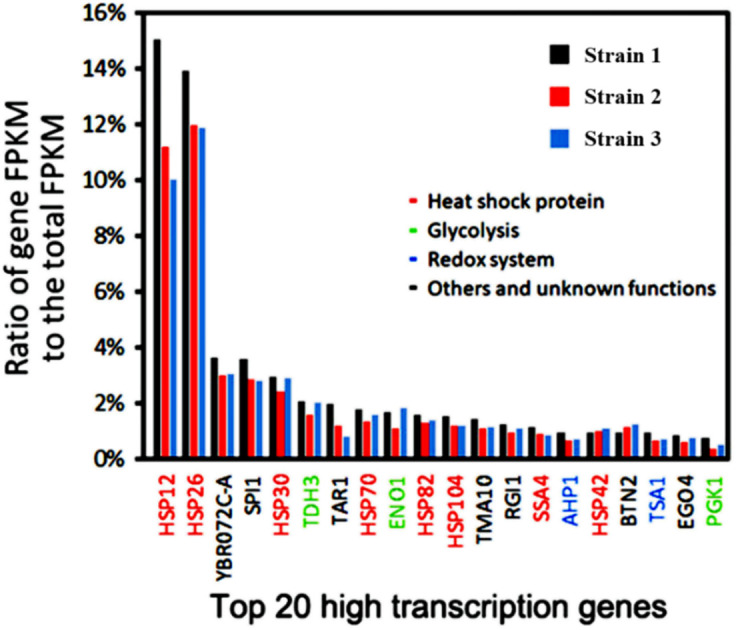

## 1. Introduction

Currently, combining microorganisms with traditional chemical manufacturing is an important approach to developing the bio-manufacturing industry for sustainable development.^1^ The traditional chemical industry has been criticized for its high energy consumption and pollution. One of the greatest challenges facing humanity in the 21st century is the energy transition. As a new, low-cost, and high-capacity industrial production model, green bio-manufacturing has emerged as a leading force in the manufacturing industry due to its advantages in sustainable development.^2^

With continuous technological innovation and progress, the product space of green bio-manufacturing has been further expanded, and the development prospects are very promising.^3^ However, there are also challenges to address. In the production process, the production strains often face disturbances in the cultivation environment and are affected by their own metabolic products, which can hinder their growth or metabolism. The tolerance of microorganisms, including their adaptability to temperature, acidity, alkalinity, substrate, and product concentrations, plays a crucial role in the biological reaction process of manufacturing bio-based chemicals.^4^ Among these factors, the thermo-tolerance of microbial cells is particularly important in determining the energy consumption and product synthesis efficiency of the fermentation process.^5^

If the temperature is not properly controlled during the later stages of microbial fermentation and metabolism, the temperature of the fermentation system can rise to above 50 °C. However, the majority of industrial microorganisms are sensitive to high temperatures, and exposure to 50 °C can result in heat shock and cell death, necessitating the use of large amounts of cooling water to control the temperature in the later stages of fermentation, leading to significant energy consumption.^6^ Antibiotics, organic acids, and amino acid products are all produced through mesophilic fermentation, with fermentation temperatures generally not exceeding 37.0 °C. During the fermentation process, microorganisms release a large amount of heat energy due to microbial growth and metabolism, causing the temperature of the fermentation broth to increase. When the fermentation temperature exceeds a certain range, the metabolism of the microbial strain accelerates, leading to premature aging, which severely affects the yield of the target metabolite. Especially in summer, the hot weather in the external environment often makes it difficult for cooling equipment to meet production requirements, leading to increased consumption of chilled water for cooling, resulting in increased energy consumption for manufacturer.^3^ The biological community is continuously exploring ways to improve the tolerance of industrial strains to reduce energy consumption.

*Saccharomyces cerevisiae*, as a core strain in industrial production, has become a research hotspot in this field.^7^ Normally, *Saccharomyces cerevisiae* cells thrive at temperatures around 30 °C. However, during the process of biological fermentation, the temperature in the fermentation tank can increase due to the heat generated by the metabolism of *Saccharomyces cerevisiae* cells and poor heat dissipation in the fermentation system. This can result in a mismatch between the optimal growth temperature and the actual fermentation temperature.^8^ The increase in fermentation temperature can lead to damage to proteins, chromosomes, cell membranes, and organelles within the *Saccharomyces cerevisiae* cells, as well as an increase in reactive oxygen species (ROS) within the cells, causing oxidative stress and affecting the activity of *Saccharomyces cerevisiae*.^9^ Research has shown that *Saccharomyces cerevisiae* undergoes changes in approximately 772–900 genes when exposed to heat stress,^10^ resulting in the upregulation of heat shock proteins (HSPs) and trehalose.^11,12^

HSPs are a group of specialized molecular chaperones synthesized by cells in response to unfavorable conditions such as heat, which help maintain protein conformation and activity. They can be classified into five major classes: HSP100, HSP90, HSP70, HSP60, and small heat shock protein (sHSP) families.^13^ HSP100 specifically induce the breakdown of protein aggregates, HSP90 assists in the folding of specific target proteins, HSP70 aids in the proper folding of newly synthesized proteins, and sHSP proteins prevent the irreversible formation of protein aggregates.^13^ HSPs are widely regarded as the most closely related functional molecules to heat resistance in cells.^14^ When organisms are subjected to heat stress, the expression level of heat shock protein mRNA can increase by 10–100 times compared to non-stress conditions.^15^ Studies have shown that the expression of HSP proteins enhances the thermotolerance of thermophilic bacteria when cultured under conditions of 55, 75, and 80 °C.^16^ HSPs are conserved proteins that are translated simply in prokaryotes and more complexly in eukaryotes. In eukaryotic cells, the transcription of HSPs is mainly regulated by heat shock transcription factors (HSFs) located upstream of the HSP genes.^17^ Under heat shock conditions, HSF forms oligomers and binds to heat shock elements (HSE), regulating the transcription and translation of HSP genes with the assistance of other factors and ATP. In non-heat shock conditions, HSF remains inactive. Hsf1 is a type of HSF that specifically regulates the transcription of genes involved in protein folding and degradation.^18,19^ The transcription of HSPs in eukaryotic cells is also linked to stress-responsive transcription factors *Msn2*/*Msn4*.

**Fig. 1:**
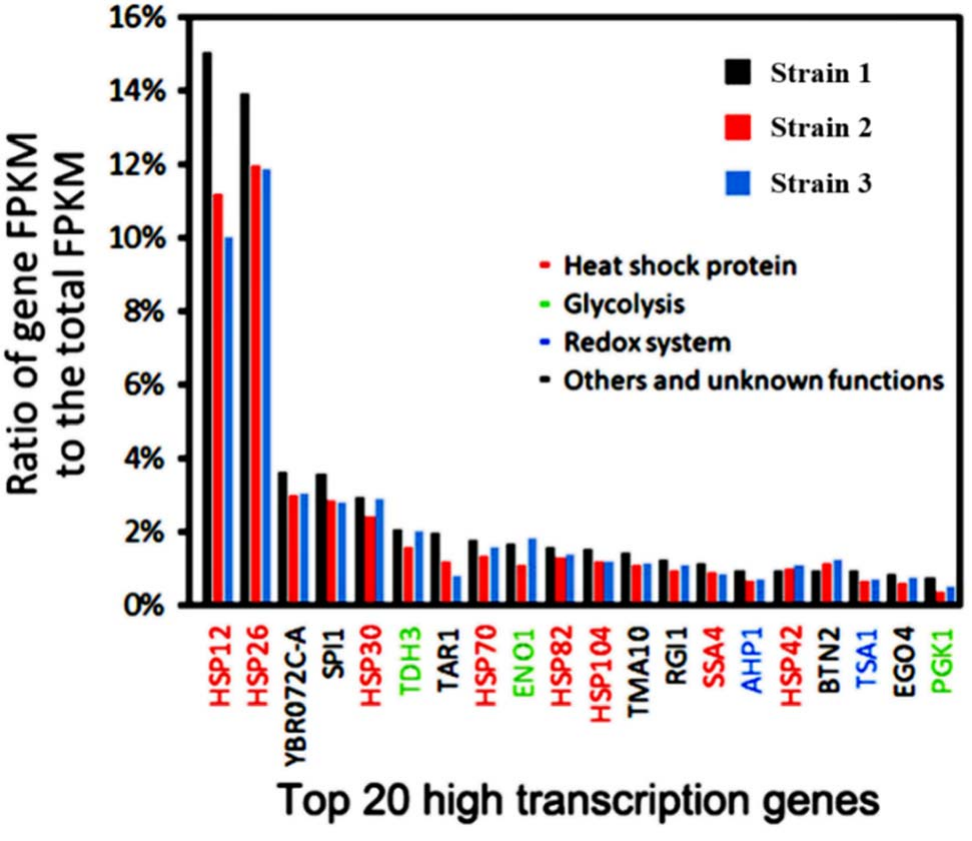
Transcriptome analysis of industrial *Saccharomyces cerevisiae* under heat stress.

Based on our previous transcriptome analysis of industrial *Saccharomyces cerevisiae* strains under heat stress conditions, it was observed that sHSP genes make up nearly half of the top 20 genes with the highest transcription levels in *Saccharomyces cerevisiae* under heat stress conditions (Fig. 1).^8^ This suggests that the sHSP naturally present in *Saccharomyces cerevisiae* are not sufficient to cope with high-temperature heat stress conditions. Adopting short-term and large-scale expression of sHSP to maintain cell survival will occupy a large amount of cell resources and have a serious impact on the expression of other proteins in the cell, reducing the production capacity of the cell.

This study selected six sHSP genes (*sHSP-HB8*, *HSP12*, *HSP30*, *HSP26*, *HSP42* and *ibpa-MB4*) from *Saccharomyces cerevisiae*, *Thermoanaerobacter tengcongensis* MB4 and *Thermus thermophiles* HB8. Standard genetic components were constructed using Golden Gate Assembly, a synthetic biology method that allows for the combination of different genetic elements. The sHSP genes were combined with either the strong promoter *TDH3*p or the weak promoter *YNL247w*p (ESI Fig. 2†) to create gene circuits. These gene circuits were then introduced into *Saccharomyces cerevisiae* BY4741 strains to verify their functions. This provides important theoretical and technical support for the development and application of microbial heat-resistant components. The research method and approach presented in this study also have significant scientific reference value for the exploration and application of other stress-resistant components. Furthermore, it plays an important role in promoting efficiency and reducing energy consumption in the biotechnology industry.

## 2. Material and methods

### 2.1. Strains, vectors and media

*Saccharomyces cerevisiae* strain BY4741 (MATa ura3Δ0 leu2Δ0 his3Δ1 met15Δ0, SY022) (Invitrogen, Carlsbad, CA) was used in our research. Engineered strains were cultured at 30 °C in SD medium lacking uracil with 20 g per L glucose. *E. coli* Top10 (Novagen, USA) competent cells were used for transformation and plasmid DNA extraction, and were cultivated at 37 °C in LB medium with 100 mg per L ampicillin or 100 mg per L kanamycin. The gene-accepting vectors HCKan-P, HCKan-O and HCKan-T for golden gate assembly were provided by Prof. Dai, Shenzhen Institutes of Advanced Technology. Plasmids used in this study are listed in (ESI Table 1†). The genes and primers were synthesized by GENEWIZ (Suzhou, China). The genomes of *Thermoanaerobe tengcongensis* MB4 and *Thermothermophiles* HB8 are all preserved in our laboratory.

### 2.2. DNA manipulation

For DNA manipulations, TIAN prep Mini Plasmid Kit (TIANGEN, China) was used to isolate plasmids from *E. coli*, respectively. Genomic DNA isolation was performed by using the TIAN amp *Saccharomyces cerevisiae* DNA Kit (TIANGEN). Enzymes used for recombinant DNA cloning and Golden Gate Assembly were purchased from Thermo Scientific (Waltham, MA) and New England Biolabs (Ipswich, MA). All of these selected genes (*HSP12*, *HSP26*, *HSP30*, *HSP42*, *sHSP-HB8*, *ibpa-MB4*) were cloned from the genomes of *Thermoanaerobe tengcongensis* MB4, *Thermothermophiles* HB8 and *Saccharomyces cerevisiae* and followed by DNA amplification using their respective primers by PCR. PCR products were purified by TIAN quick Midi Purification Kit (TIANGEN) and genetic circuits were constructed with standard vector parts by employing the Golden Gate Assembly.^20^ The candidate sHSP genes were ligated into the POT vectors along with promotor upstream and *SLM5t* terminator downstream, followed by transformation into *Saccharomyces cerevisiae* strain BY4741. Primers used in the DNA assembly are summarized in (ESI Table 2†).

### 2.3. Real time reverse transcription PCR

The total RNA was extracted from *Saccharomyces cerevisiae* cells by Trizol and served as the template to obtain complementary DNA using the TransScript First-Strand cDNA Synthesis Kit (Trans, China). The converted cDNA and the specific primers was added to Top/Tip Green qRCR SuperMix to subject RT-PCR analysis employing the Roche LightCycler 96 Real-Time PCR System (Cal, US). ACT1 was selected as the internal reference gene.

### 2.4. Shake-flask cultivation

The engineered strains were grown at 42 °C for high temperature stress in SD medium lacking uracil with 20 g per L glucose. Seed cultures were prepared by growing all strains individually in 15 mL culture tubes containing 2 mL medium at 30 °C and 200 rpm overnight. Flasks (250 mL) containing 30 mL medium were then inoculated with the resulting seed cultures to an OD_600_ of 0.1. The strains were grown for 72 hours, and OD_600_ was measured every 12 hours using a spectrophotometer, model U-2900 (HITACHI, Chiyoda, Tokyo). All data represent the mean standard deviation from three independent experiments.

### 2.5. Cell viability analysis of engineered strains

A serial dilution assay was completed by taking samples at 24 h and serially diluting them 10-fold, followed by spotting 2.5 μL of the dilutions onto SD medium lacking uracil with 20 g per L glucose plates. The thermo tolerance ability of the engineered strains was characterized.

### 2.6. The enzyme activities assays of pyruvate kinase (PK) and malate dehydrogenase (MDH)

The culture method described in Section 2.4 was applied. After 72 h, cells were harvested by centrifugation, and the supernatant was discarded. Cells was washed several times in 100 mM of Tris–HCl (pH 7.0) buffer and suspended in extraction buffer (100 mM of Tris–HCl, pH 7.0, 1 m of MDTT, 10 mM of MgCl_2_, 1 mM of EDTA), then disrupted using ultrasonication (work 2 s, intermittent 2 s, duration 10 min, power 80 W) in an ice bath. After the mixture was centrifuged for 5 min at 4 °C, 8000 rpm, most of the crude enzyme was in the supernatant. Pyruvate kinase (PK) and malate dehydrogenase (MDH) were assayed using commercial kits (Jian Cheng Biotech Company, Nanjing, China) following the manufacturers' instructions.

## 3. Results and discussion

### 3.1. Construction of engineered strains

Currently, it is widely believed that HSP are closely related to cellular thermo-tolerance. They can stabilize cell structures and ensure the normal physiological functions of cells. When an organism is subjected to heat stress, the expression level of heat shock protein mRNA can be 10–100 times higher than that under non-stress conditions.

**Fig. 2:**
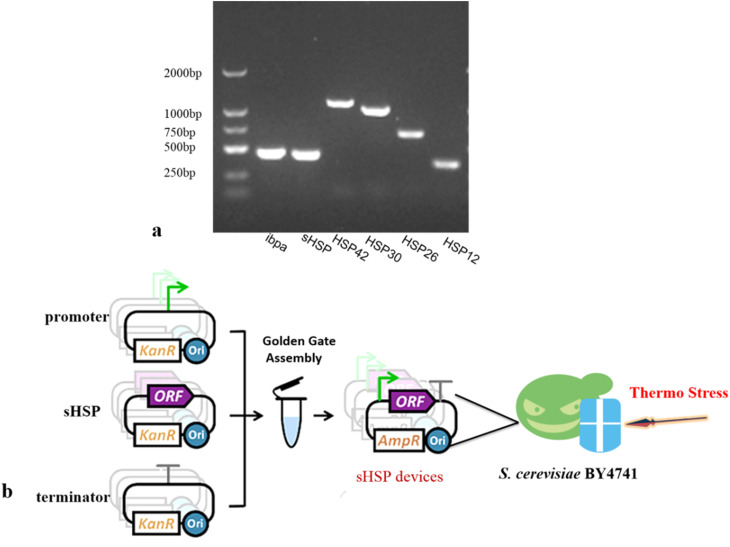
Construction sHSP devices using Golden Gate Assembly. (a) Agarose gel electrophoresis of sHSP genes (b) construction strategy.

*Saccharomyces cerevisiae* has three major sHSP: *HSP26*, *HSP12*, and *HSP30*. sHSPs have certain common structural and regulatory characteristics. They are induced by heat shock and various developmental processes. They have the unusual ability to assemble into high molecular weight complexes called Heat Shock Granules (HSGs). Based on this conclusion and previous research results, we selected four sHSP genes from *Saccharomyces cerevisiae* (*HSP12*, *HSP26*, *HSP30*, and *HSP42*), as well as the sHSP gene *ibpa-MB4* from *Thermoaerobe tengcongensis* MB4 and *sHSP-HB*8 from *Thermothermophiles* HB8. We used the three types of microorganisms genome as a template for gene PCR amplification, and successfully amplified all sHSP standard elements (Fig. 2a).

To precisely regulate the expression of sHSP, we combined the sHSP genes with either the strong promoter *TDH3*p or the weak promoter *YNL247w*p (ESI Fig. 2†) to construct gene circuits in the expression vectors POT1 (Fig. 2b). These constructed vectors were then transformed into the *Saccharomyces cerevisiae* BY4741 strain. As a result, we obtained 12 genetically engineered strains. The control strain used in our experiments was the *Saccharomyces cerevisiae* BY4741 strain transformed with empty vectors POT1. The RT-PCR results (ESI Fig. 2†) showed that the engineered strains were successfully constructed and can be used for later experiments.

### 3.2. Characterization of sHSP devices in *Saccharomyces cerevisiae*

**Fig. 3:**
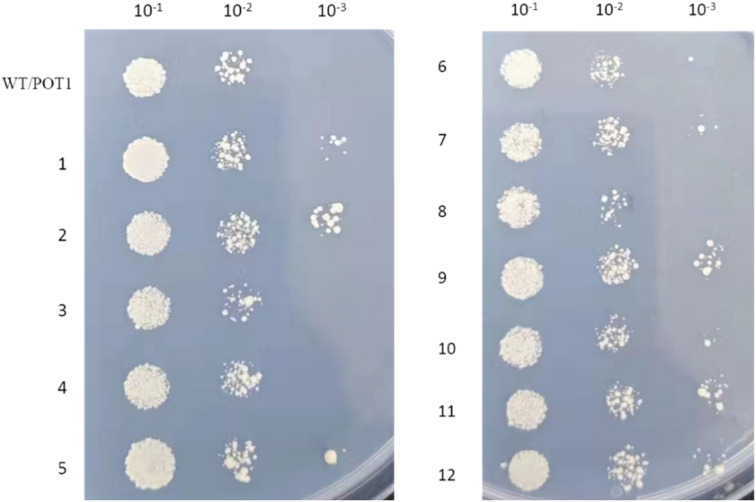
Cell viability.

We assigned numbers to the engineered strains (Table 1), and these strains were cultivated under high temperature stress at 42 °C. First, we conducted a cell viability test, and the results indicated (Fig. 3) that, except for engineered strains 3, 4, and 8, all the other engineered strains exhibited improved heat resistance compared to the control strain.

**Table 1:** Engineered strains number

Strains number	Promoter	Gene
1	*TDH3*p	*HSP12*
2	*TDH3*p	*HSP26*
3	*TDH3*p	*HSP30*
4	*TDH3*p	*HSP42*
5	*TDH3*p	*sHSP-HB8*
6	*TDH3*p	*ibpa-MB4*
7	*YNL247w*p	*HSP12*
8	*YNL247w*p	*HSP26*
9	*YNL247w*p	*HSP30*
10	*YNL247w*p	*HSP42*
11	*YNL247w*p	*sHSP-HB8*
12	*YNL247w*p	*ibpa-MB4*

**Fig. 4:**
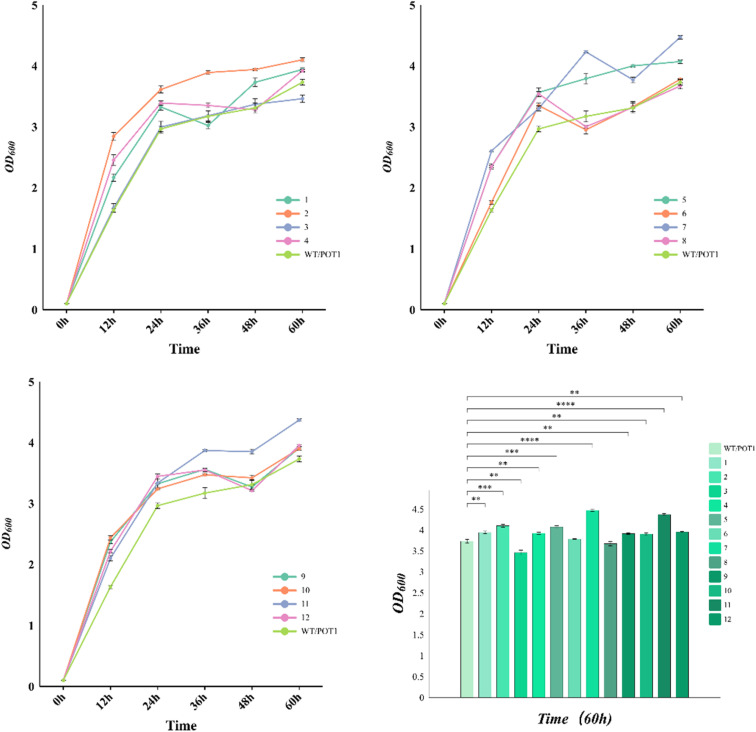
Cell growth of engineered strains.

In the growth curve observed at 42 °C (Fig. 4), it is evident that the engineered strains, except for 3 and 8, exhibited improved growth compared to the control strain. Specifically, at 72 hours, the cell densities of strains 7 and 11, which were the top-performing engineered strains, increased by 19.8% and 17.2%, respectively, in comparison to the control strain. It is worth noting that the gene circuits in these two engineered strains are regulated by the weak promoter *YNL247w*p, indicating that moderate maintenance of sHSP expression has a better effect on the thermo-tolerance of the engineered strains.

### 3.3. The key enzyme activity of engineered strains

sHSP plays a crucial role in preventing the aggregation of heat-damaged proteins and facilitating their refolding after heat shock. To verify the changes in enzyme activity, we selected key enzymes in *Saccharomyces cerevisiae* cells. Pyruvate kinase (PK) is a major rate-limiting enzyme in glycolysis and its activity reflects the physiological condition of cells. Malate dehydrogenase (MDH), on the other hand, is a key enzyme in the TCA cycle that catalyzes the dehydrogenation of malate oxaloacetate and plays a significant role in energy metabolism.

**Fig. 5:**
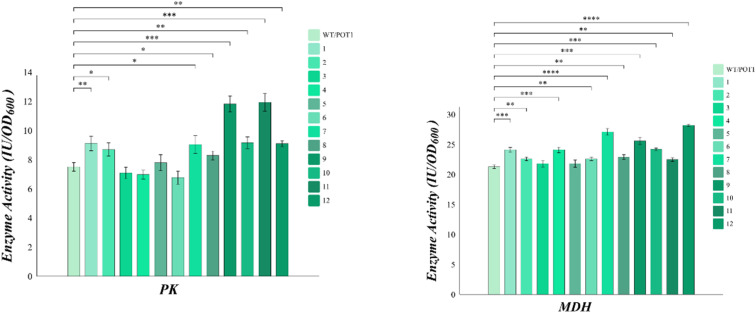
The key enzyme activity of engineered strains.

The analysis of Fig. 5 shows that, apart from engineered strains 3, 4, and 6, all the other engineered strains exhibited higher pyruvate kinase (PK) activities compared to the control. However, it is worth noting that engineered strain 8 displayed poor growth at high temperatures, despite showing higher pyruvate kinase (PK) activities than the control. On the other hand, the malate dehydrogenase (MDH) activities of all the engineered strains were higher than that of the control. Notably, engineered strains 7 and 11 demonstrated malate dehydrogenase (MDH) activities that were 27.2% and 34.4% higher than the control, respectively.

## 4. Conclusion

This study aimed to obtain thermo-tolerance *Saccharomyces cerevisiae* strains as the design concept, using synthetic biology methods to construct small HSP gene circuits with precise regulation of expression by selecting promoters of different strengths and validating their functionality. As a result, engineered strains with improved thermo-tolerance were obtained. Although the heat resistance mechanism of the small HSP gene needs further investigation, this study provides important theoretical and technical support for the development and application of thermo-tolerance genetic elements in microorganisms. Further research can be conducted from the following aspects: our results indicate that small HSPs contribute to maintaining cellular protein homeostasis. It is possible to explore key protective substances and functional proteins involved in the metabolic pathways related to maintaining protein balance within cells, with the expectation of further improving the heat resistance of *Saccharomyces cerevisiae*; under heat stress, damage can occur to the yeast's cell membrane, DNA, and other aspects. Repairing cell damage under heat stress can be approached from multiple angles; currently, the promoters our uesd are constitutive and expressed even at normal temperatures, which burdens the cells. It is hoped to discover effective heat-inducible promoters to achieve real-time expression of components. Furthermore, the assembly of heat shock protein devices at different intensities can be used to achieve a cascade heat-resistant regulation in brewing yeast. The research methodology and approach also have significant scientific reference value in the exploration and application of other stress-resistant genetic elements, playing an important role in promoting efficient and low-energy consumption in the biotechnology industry.

## Author contributions

Mei-ling Zhang: methodology, construction and fermentation validation of engineered strains, writing – review & editing. Hui Zhang: construction and fermentation validation of engineered strains, writing – review & editing. Ya-xin He: helped with DNA and genetic manipulations and fermentation experiments. Zhao-hui Wu: methodology, writing – review & editing. Ke Xu: methodology, writing – review & editing.

## Conflicts of interest

We declare that we have no financial and personal relationships with other people or organizations that can inappropriately influence our work, there is no professional or other personal interest of any nature or kind in any product, service and/or company that could be construed as influencing the position presented in, or the review of, the manuscript entitled.

## Supplementary Material

RA-013-D3RA05216H-s001
