# Supplementary material for: Improving thermo-tolerance of Saccharomyces cerevisiae by precise regulation of the expression of small HSP
Source: RSC Adv. 2023 Dec 12;13(51):36254–60. doi: 10.1039/d3ra05216h (PMC10714670; doi:10.1039/d3ra05216h)
Supplement: RA-013-D3RA05216H-s001 [file RA-013-D3RA05216H-s001.pdf]

Electronic Supplementary Material (ESI) for RSC Advances.
This journal is © The Royal Society of Chemistry 2023

Supplementary material

Improving thermo-tolerance of *Saccharomyces cerevisiae* by precise regulation the expression of small HSP

Mei-ling Zhang[1], Hui Zhang[1],Ya-xin He[1], Zhao-hui Wu[1], Ke Xu[1,2]*

1. Department of Life Science, Tangshan Normal University, Tangshan , 063000, PR China
2. Key Lab for Industrial Biocatalysis, Ministry of Education, Department of Chemical Engineering, Tsinghua University, Beijing, 100084, PR China

Table S1 Plasmids used in this study

Plasmid	Host Strains	Reference
HCKan-P	*E.coli*	Guo et al., 2015
HCKan-O	*E.coli*	Guo et al., 2015
HCKan-T	*E.coli*	Guo et al., 2015
POT-1	*E.coli and S. cerevisiae*	Guo et al., 2015

Table S2 Primers used in this study

Gene	Primer-F	Primer-R
HSP12	AGCGTGGGTCTCAGATG TCTGACGCAGGTAGAAA	GTGCTGGGTCTCGGCTA CTTCTTGGTTGGGTCTTCTT
HSP26	AGCGTGGGTCTCAGATG TCATTTAACAGTCCATTTTT	GTGCTGGGTCTCGGCTA GTTACCCCACGATTCTTGAG
HSP30	AGCGTGGGTCTCAGATG AACGATACGCTATCAAGCTT	GTGCTGGGTCTCGGCTA AGCAGTATCTTCGACAGCTT
HSP42	AGCGTGGGTCTCAGATG AGTTTTTATCAACCATCCCT	GTGCTGGGTCTCGGCTA ATTTTCTACCGTAGGGTTGG
sHSP-HB8	AGCGTGGGTCTCAGATG CTGGAGCGCCACGACCGCCT	GTGCTGGGTCTCGGCTA CGCCTCCTTTAGGGGAAGGG
ibpa-MB4	AGCGTGGGTCTCAGATG AGCTTAATGAGAAGAGGAAG	GTGCTGGGTCTCGGCTA CTCTATATCAATTCTTCTTT

Table S3 The information about TDH3 and YNL247 genes and promoters

**Gene**	Gene sequence	Information description
TDH3	1 ATGGTTAGAG TTGCTATTAA CGGTTTCGGT AGAATCGGTA GATTGGTCAT GAGAATTGCT<br>61 TTGTCTAGAC CAAACGTCGA AGTTGTTGCT TTGAACGACC CATTCATCAC CAACGACTAC<br>121 GCTGCTTACA TGTTCAAGTA CGACTCCACT CACGGTAGAT ACGCTGGTGA AGTTTCCCAC<br>181 GATGACAAGC ACATCATTGT CGATGGTAAG AAGATTGCTA CTTACCAAGA AAGAGACCCA<br>241 GCTAACTTGC CATGGGGTTC TTCCAACGTT GACATCGCCA TTGACTCCAC TGGTGTTTTC<br>301 AAGGAATTAG ACACTGCTCA AAAGCACATT GACGCTGGTG CCAAGAAGGT TGTTATCACT<br>361 GCTCCATCTT CCACCGCCCC AATGTTCGTC ATGGGTGTTA ACGAAGAAAA ATACACTTCT<br>421 GACTTGAAGA TTGTTTCCAA CGCTTCTTGT ACCACCAACT GTTTGGCTCC ATTGGCCAAG<br>481 GTTATCAACG ATGCTTTCGG TATTGAAGAA GGTTTGATGA CCACTGTCCA CTCTTTGACT<br>541 GCTACTCAAA AGACTGTTGA CGGTCCATCC CACAAGGACT GGAGAGGTGG TAGAACCGCT<br>601 TCCGGTAACA TCATCCCATC CTCCACCGGT GCTGCTAAGG CTGTCGGTAA GGTCTTGCCA<br>661 GAATTGCAAG GTAAGTTGAC CGGTATGGCT TTCAGAGTCC CAACCGTCGA TGTCTCCGTT<br>721 GTTGACTTGA CTGTCAAGTT GAACAAGGAA ACCACCTACG ATGAAATCAA GAAGGTTGTT<br>781 AAGGCTGCCG CTGAAGGTAA GTTGAAGGGT GTTTTGGGTT ACACCGAAGA CGCTGTTGTC<br>841 TCCTCTGACT TCTTGGGTGA CTCTCACTCT TCCATCTTCG ATGCTTCCGC TGGTATCCAA<br>901 TTGTCTCCAA AGTTCGTCAA GTTGGTCTCC TGGTACGACA ACGAATACGG TTACTCTACC<br>961 AGAGTTGTCG ACTTGGTTGA ACACGTTGCC AAGGCTTAA	Glyceraldehyde-3-phosphate dehydrogenase (GAPDH)
	1 ATGAATATCT TCATAAAAGC CCTGAGAAGA TATACTATAA TGTCTACGCC GAAGATTGTG<br>61 CAGCCCAAAT GGAAGGTTCC AACGCCACAA GCTAAAGAAA CTGTGTTGAA GTTGTACAAC	Cysteinyl-tRNA synthetase

121 AGTTTAACAA GATCTAAGGT TGAATTCATT
CCGCAATCTG GCAATAGAGG TGTCACTTGG
181 TACTCTTGCG GTCCTACTGT TTACGATGCC
TCCCATATGG GTCATGCCAG AAACTATGTC
241 TCTATTGATA TCAATAGAAG AATTATTCAA
GATTATTTTG GTTACGACGT GCAATTTGTG
301 CAAAATGTTA CTGATATCGA CGATAAAATT
ATTTTGAGAG CTAGACAAAA CTATTTATTT
361 GACAATTTTG TCAAAGAAAA TGATACCAAA
TTCAACGCCA CTGTTGTTGA CAAGGTCAAA
421 ACCGCACTTT TCCAATATAT CAACAAAAAT
TTTACTATTC AAGGCAGCGA GATCAAAACT
481 ATCGAAGAAT TTGAAACTTG GTTATCGAAT
GCTGATACTG AAACTTTAAA ATTGGAGAAT
541 CCTAAATTCC CTATGCATGT CACCGCAGTT
CAAAATGCTA TTGAATCAAT CACTAAGGGC
601 GATTCCATGG ACGCAGAAGT TGCCTTTGAA
AAAGTCAAGG ACGTTACGGT TCCTCTATTG
661 GATAAAGAAT TGGGCTCTAC CATTAGCAAT
CCAGAGATTT TCCGCCAACT TCCAGCTTAC
721 TGGGAACAGA AATTCAATGA TGACATGTTA
TCATTAAACG TGCTACCTCC CACCGTTACA
781 ACTCGTGTTT CTGAGTACGT TCCAGAAATT
ATTGACTTTG TTCAAAAAAT TATTGATAAT
841 GGTTACGCAT ATGCCACTTC CGACGGTTCC
GTGTACTTTG ATACTTTAAA ATTTGACAAA
901 TCCCCAAATC ATGACTATGC TAAATGCCAG
CCATGGAATA AGGGCCAGTT AGACTTAATT
961 AATGATGGTG AAGGGTCCTT AAGCAACTTT
GCTGATAACG GAAAAAAGTC GAATAATGAT
1021 TTTGCTTTAT GGAAGGCTTC CAAGGCAGGT
GAACCTGAGT GGGAATCACC ATGGGGTAAG
1081 GGTAGACCAG GATGGCATAT TGAATGTTCT
GTGATGGCCA GTGATATCCT AGGCTCTAAC
1141 ATCGATATTC ATTCAGGTGG CATCGATTTG
GCCTTTCCTC ACCATGATAA CGAATTGGCT
1201 CAATCCGAGG CTCGCTTCGA CAATCAACAG
TGGATCAACT ATTTCTTACA TACGGGCCAT
1261 TTACATATTG AGGGTCAAAA AATGTCTAAA
TCCTTAAAGA ATTTCATTAC CATTCAAGAA
1321 GCTTTGAAAA AATTCTCACC GCGCCAATTA
AGATTGGCTT TTGCCTCAGT ACAATGGAAC
1381 AATCAATTGG ATTTCAAGGA ATCTTTGATC
CATGAAGTAA AGTCATTTGA AAACTCCATG

1441 AACAATTTTT TTAAGACTAT TAGAGCATTG
AAGAACGATG CAGCTTCTGC AGGTCATATC
1501 TCTAAAAAGT TTAGTCCCTT AGAGAAAGAA
TTATTGGCTG ATTTTGTTGA AAGTGAATCG
1561 AAAGTCCATT CGGCGTTCTG TGATAATTTA
TCCACACCTG TTGCTTTGAA GACACTGAGC
1621 GAATTAGTGA CCAAGTCAAA CACATACATT
ACCACTGCAG GTGCTGCTTT AAAAATTGAG
1681 CCCTTGATTG CTATCTGTAG CTACATCACC
AAAATCTTAA GAATAATTGG ATTTCCATCC
1741 CGTCCTGACA ATTTGGGTTG GGCAGCCCAA
GCTGGCTCCA ACGATGGATC CCTAGGCTCA
1801 TTGGAAGACA CTGTTATGCC ATATGTTAAG
TGTTTATCCA CATTTAGAGA TGATGTACGT
1861 TCCTTAGCTA TCAAGAAAGC CGAACCCAAG
GAATTCTTGC AATTAACGGA TAAAATTAGA
1921 AACGAAGATT TGCTAAACTT GAATGTTGCC
TTGGATGATA GGAATGGACA ATCTGCCTTG
1981 ATCAAATTTT TGACTAACGA TGAAAAATTG
GAAATTGTCA AGCTAAACGA GGAGAAACAT
2041 GCCAACGAAC TAGCAAAGAA ACAAAAGAAA
TTGGAACAGC AGAAATTAAG AGAGCAGAAG
2101 GAAAACGAGA GGAAGCAGAA AGCTCAAATT
AAACCACAAG ATATGTTCAA GGATGTCACA
2161 TTGTACAGTG CTTGGGACGA GCAAGGCCTT
CCAACAAAGG ACAAAGACGG TAATGATATC
2221 ACCAAGAGTA TGACCAAGAA GTTGAAGAAG
CAATGGGAAC AACAAAAGAA GCTACATGAA
2281 GAGTACTTTG GTGAAGACAA ATAG
1 ATAAAAAACA CGCTTTTTCA GTTCGAGTTT constitutive promoter
ATCATTATCA ATACTGCCAT TTCAAAGAAT
61 ACGTAAATAA TTAATAGTAG TGATTTTCCT
AACTTTATTT AGTCAAAAAA TTAGCCTTTT
121 AATTCTGCTG TAACCCGTAC ATGCCCAAAA
TAGGGGGCGG GTTACACAGA ATATATAACA
181 TCGTAGGTGT CTGGGTGAAC AGTTTATTCC
TGGCATCCAC TAAATATAAT GGAGCCCGCT
241 TTTTAAGCTG GCATCCAGAA AAAAAAAGAA
TCCCAGCACC AAAATATTGT TTTCTTCACC
301 AACCATCAGT TCATAGGTCC ATTCTCTTAG
CGCAACTACA GAGAACAGGG GCACAAACAG
TDH3p 361 GCAAAAAACG GGCACAACCT CAATGGAGTG
ATGCAACCTG CCTGGAGTAA ATGATGACAC
421 AAGGCAATTG ACCCACGCAT GTATCTATCT

	CATTTTCTTA CACCTTCTAT TACCTTCTGC<br>481 TCTCTCTGAT TTGGAAAAAG CTGAAAAAAA<br>AGGTTGAAAC CAGTTCCCTG AAATTATTCC<br>541 CCTACTTGAC TAATAAGTAT ATAAAGACGG<br>TAGGTATTGA TTGTAATTCT GTAAATCTAT<br>601 TTCTTAAACT TCTTAAATTC TACTTTTATA<br>GTTAGTCTTT TTTTTAGTTT TAAAACACCA<br>661 AGAACTTAGT TTCGAATAAA CACACATAAA<br>CAAACAAA	
YNL247wp	1 TACTAGATAA AGTGCAAAAG GTTGATAACG<br>ACTGCAATAG TGGTAGCAAC TTAAAAAGCC<br>61 CTCCTAATAT CATTAGTGTT CATGCAAGAA<br>TACTTACTAT TAAAAATGAG ATGGAAAATT<br>121 TCAGCTCATC GCAATAAAAA ATTTTCAGCG<br>CGGGTGACCG CAAACATTTT TCATCGCGAA<br>181 GAGTCAAAAG ACATTATCAA TTGAAAAGAA<br>CAGTATTGCT AATTGGCGTC GGTGATTAGG<br>241 TGTCCGGTCA AGTTCTTATT CTATGC	constitutive promoter

Table S4 Primers used for qPCR analysis

Gene	Primer-F	Primer-R
HSP12	CGCAGGTAGAAAAGGATTCGG	TTGGGTCTTCTTCACCGTGG
HSP26	AAGAGGCTACGCACCAAGAC	ACCATCCTTCTGAGGCTTCA
HSP30	CTGGTTCTTGTCCTGGCCAT	TGTTTCAGGAGCCGCATCTT
HSP42	AGGACCAACCAACAGGCAAA	AGCACGGGGAATTTAACGGT
sHSP-HB8	CGCCTGGAAACCCTGAGAAA	CTTTAGGGGAAGGGCCGTG
ibpa-MB4	AAGAGGAAGAGACTGGTGGGA	TCTTTGGCTTGCTCGGATGG

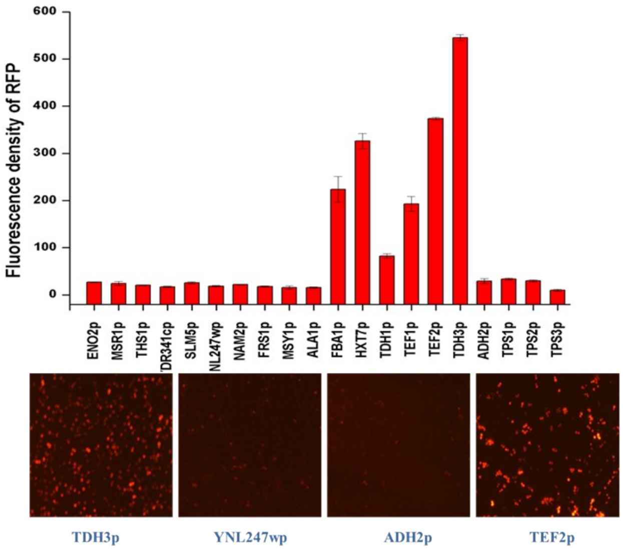

Figure S1 Characterization of promoter strength

Experimental method of Figure S1

All promoters followed by DNA amplification using their respective primers by PCR. PCR products were purified by TIAN quick Midi Purification Kit (TIANGEN) and genetic circuits were constructed with standard vector parts by employing the Golden Gate Assembly. The promoters were ligated into the POT vectors as promotor-GFP-SLM5t, followed by transformation into *Saccharomyces cerevisiae* strain BY4741. The engineered strains were grown at 30℃,12h in SD medium lacking uracil with 20 g/L glucose, then we detect the intensity of red fluorescence using a fluorescence microplate reader and observe it under a fluorescence microscope, the excitation wavelength 532nm (green light), emission wavelength 588nm.

a

b

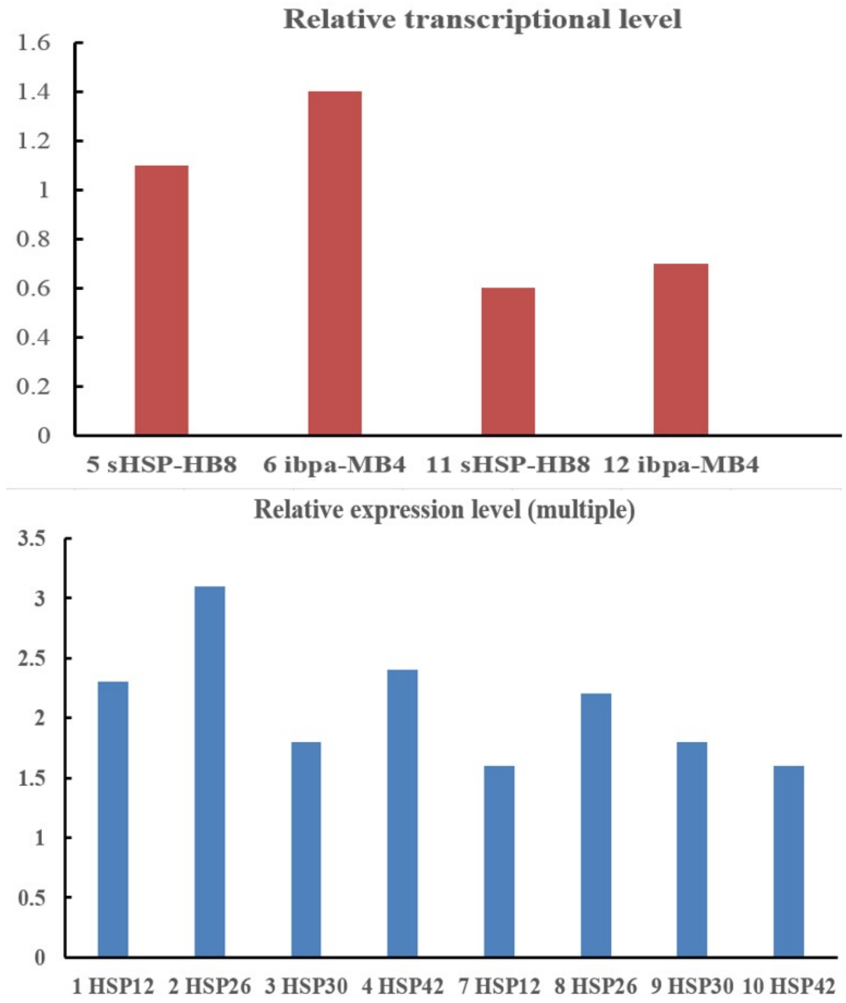

Figure S2 Transcriptional levels of genetic circuits by RT-qPCR. (a) ***ibpa-MB4 and sHSP-HB8; (b) HSP12, HSP26, HSP30, and HSP42 (*** b. The expression of each gene relative to the expression of the same gene in the wt strain. The calculation method is to divide the expression level of HSP genes in the engineered strain by the expression level of HSP genes in the wild-type strain ***).***

Experimental method of Figure S2

The total RNA was extracted from *Saccharomyces cerevisiae* cells by Trizol and served as the template to obtain complementary DNA using the TransScript First-Strand cDNA Synthesis Kit (Trans, China). The converted cDNA and the specific primers was added to Top/Tip Green qRCR SuperMix to subject RT-PCR analysis employing the Roche LightCycler 96 Real-Time PCR System (Cal, US). ACT1 was selected as the internal reference gene.

Supplementary references

1. Guo Y, Dong J, Zhou T, et al. YeastFab: the design and construction of standard

biological parts for metabolic engineering in *Saccharomyces cerevisiae*. Nucleic Acids Res, 2015, 43(13):e88.
